# Effects of ASMR on mental fatigue recovery revealed by EEG power and brain network analysis

**DOI:** 10.3389/fnhum.2025.1619424

**Published:** 2025-07-16

**Authors:** Yueguang Si, Yu Sun, Kuijun Wu, Lingyun Gao, Sujie Wang, Mengru Xu, Xuchen Qi

**Affiliations:** ^1^Shaoxing People's Hospital (Shaoxing Hospital, Zhejiang University School of Medicine), Shaoxing, China; ^2^The State Key Laboratory for Brain-Computer Intelligence, and the Key Laboratory for Biomedical Engineering of Ministry of Education of China, Department of Biomedical Engineering, Zhejiang University, Hangzhou, China; ^3^Department of Neurosurgery, Shaoxing People's Hospital, Zhejiang University School of Medicine, Hangzhou, China; ^4^College of Mathematical Medicine, Zhejiang Normal University, Jinhua, China; ^5^The Key Laboratory for Biomedical Engineering of Ministry of Education of China, Department of Biomedical Engineering and Instrument Science, Zhejiang University, Hangzhou, China; ^6^The School of Electrical and Information Engineering, Zhengzhou University, Zhengzhou, China; ^7^Henan Key Laboratory of Brain Science and Brain Computer Interface Technology, Zhengzhou, China; ^8^The Department of Neurosurgery, Sir Run Run Shaw Hospital, Zhejiang University School of Medicine, Hangzhou, Zhejiang, China

**Keywords:** mental fatigue, autonomous sensory meridian response (ASMR), psychomotor vigilance task (PVT), mental fatigue recovery, functional brain connectivity

## Abstract

**Introduction:**

Mental fatigue, resulting from prolonged cognitive tasks or sleep deprivation, significantly impacts safety and performance, particularly in high-risk environments. However, effective intervention methods are limited, highlighting the urgent need for new approaches to alleviate mental fatigue. This study explores the effectiveness of Autonomous Sensory Meridian Response (ASMR) as a novel intervention for alleviating mental fatigue.

**Methods:**

A within-subject design was employed in this work, where 28 healthy young subjects (M/F = 17/11, age = 21.82 ± 0.37 years) were requested to perform a continuous 30 min sustained attention task (named No-Break session) and a 30 min task with a 4-min mid-task ASMR break (named ASMR-Break session) at a counterbalanced order. The immediate effect and general effect of ASMR were quantitatively assessed on behavioral performance and EEG characteristics.

**Results:**

Behaviorally, only significant immediate effect was revealed as showing in reduced reaction time. Further interrogation of brain dynamics showed complex patterns of spatio-spectrum alterations and an interaction in small-world metric in theta band. Specifically, the ASMR intervention prevented an increase in small-worldness, and the correlation between changes in small-worldness and reaction times diminished after the intervention.

**Discussion:**

In sum, this preliminary investigation provides insight into ASMR's neural mechanisms and suggests it may help attenuate fatigue. Further research in larger, more diverse samples will be necessary to confirm its utility for mental fatigue management in real-world settings.

## 1 Introduction

Heuristically, mental fatigue is characterized by subjective mental exhaustion, often caused by prolonged work or insufficient sleep. This condition can lead to decreased alertness and reduced attention to tasks (Zhao et al., 2012), potentially resulting in severe consequences such as fatigue driving (Zhao et al., 2016) and low productivity in working environment (Uehli et al., 2014). In fact, 21% of global annual traffic accidents were found to be attributed to driving fatigue (Vivoli et al., 2006). In Canada, more than 50% of drivers were driving under fatigue and 20% were driving even in semi-sleep (Vingilis et al., 2005); while this ratio was 40% in China according to the China Expressway Network. To reduce these undesirable consequences, understanding the neural mechanisms underlying mental fatigue is crucial for developing effective detection and management strategies, improving work efficiency, and reducing potential hazards in high-stakes environments.

Despite extensive research on mental fatigue, studies on interventions to alleviate it remain relatively scarce. Common methods include rest (Gui et al., 2015), listening to music (Taya et al., 2018), and exercising (Xu et al., 2017). However, these methods have certain limitations. For example, although many studies have reported the effectiveness of rest (Chen et al., 2010; Blasche et al., 2018), mental fatigue does not necessarily recover after rest (Jacquet et al., 2021) and is dependent on the characteristics of the rest (i.e., duration of the rest, when to administer the rest, etc.; Tait et al., 2024; Lim and Kwok, 2016). Listening to music has long been recognized to be associated with positive emotions (McCraty et al., 1998), which may in return alleviate mental fatigue through modulating the motivation. Nevertheless, the impact of music on mental fatigue can vary based on personal preferences and the specific characteristics of the music (McCraty et al., 1998). Similarly, the effects of exercise on mental fatigue can vary widely depending on the type, intensity, and duration of both the mental and physical activities involved (Holgado et al., 2020). In fact, we previously reported that a 15-min cycling exercise failed to exhibit better recovery in comparison with rest (Gao et al., 2022). Therefore, it is particularly important to find a more effective and universally applicable intervention for mental fatigue recovery.

Autonomous sensory meridian response (ASMR) is a pleasurable, head-orientated tingling sensation that is typically in response to specific audiovisual stimuli (i.e., whispers, soft sounds, tapping, etc.; Barratt and Davis, 2015). Despite its non-scientific origins, substantial interests and apparent prevalence were gained on ASMR-related applications. Of note, the somatosensory “tingles” are associated with a reduction in heart rate and an increase in skin conductance responses (Poerio et al., 2018). More importantly, recent studies have revealed positive effect during ASMR, including a feeling of relaxation (Barratt and Davis, 2015) and alleviation of anxiety (Eid et al., 2022), indicating the potential role of ASMR as a novel intervention for mental fatigue.

Taking all the above into consideration, the effects of ASMR intervention on mental fatigue recovery were comprehensively assessed in the current work. Specifically, the work includes a within-subject design comparison of two sessions, that is, No-Break: comprising a continuous 30-min sustained attention task [i.e., Psychomotor Vigilance Task (PVT)], and ASMR-Break: consisting of two 15-min PVT tasks with a 4-min ASMR break in between. The behavior performance in terms of reaction time as well the brain dynamics [as measured in terms of power spectral density (PSD) and brain network metrics] were then quantitatively examined to provide a comprehensive assessment of the effects on fatigue recovery. To the best of our knowledge, this is the first study to explore the effect of ASMR on fatigue recovery and more importantly the underlying neural mechanisms. Based upon previous studies on ASMR (Barratt and Davis, 2015), we hypothesized that ASMR would lead to beneficial effect on mental fatigue. Moreover, based upon prior neuroimaging studies of brain activities during ASMR (Swart et al., 2022; Smith et al., 2017; Fredborg et al., 2021), we further hypothesized that ASMR would lead to complex spatio-spectral patterns as seen in both PSD and functional brain network reorganization.

## 2 Materials and methods

### 2.1 Subjects

In this study, 28 healthy university students (males/females = 17/11, age = 21.82 ± 0.37 years) were recruited from Zhejiang University. The same self-administered questionnaire was performed and the ASMR audio was played in a quiet room prior the experiment to ensure participants met the inclusion criteria: (1) no discomfort from the ASMR audio; (2) absence of chronic physical or psychosocial illnesses; (3) no history of significant somatic diseases; (4) have slept for at least 7 h the night prior to the experiment; (5) no intake of alcohol and caffeine on the day of the experiment. After being informed of the experimental protocol, subjects completed a consent form based on the Helsinki Declaration. Ethical approval for this study was obtained from the Institutional Review Board of Zhejiang University (IRB no. [2022]-47).

### 2.2 Experimental protocol

**Figure 1:**
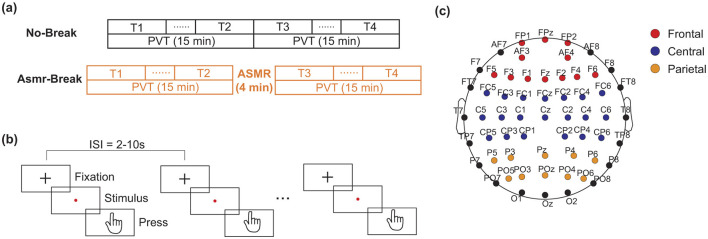
Schematic of the experimental paradigm. **(a)** The No-Break session consists of a continuous 30-min PVT), whereas the ASMR-Break session includes a 4-min Autonomous Sensory Meridian Response (ASMR) intervention inserted into the PVT. Time points T1 and T4 represent the first and last 6-min of task engagement, respectively. Time points T2 and T3 indicate the 6-min segments immediate before and after the ASMR intervention period. **(b)** Protocol of the PVT. **(c)** The assignment of electrodes in frontal, central, and parietal cortex.

This study employed a within-subject design to investigate the impact of ASMR on mental fatigue recovery. Specifically, each participant completed two sessions (named No-Break and ASMR-Break session respectively) in a counterbalanced manner with a minimum interval of 5 days between the two sessions to ensure sufficient recovery from fatigue (Figure 1). A 30-min PVT was used to induce a substantial fatigue effect, as described in our previous research (Gao et al., 2020; Sun et al., 2014). In brief, participants were instructed to monitor a screen and promptly press a button upon the appearance of a red dot. The inter-trial interval varied randomly between 2 and 10 seconds (mean = 6 s). In the *No-Break* session, subjects were required to perform the 30-min PVT continuously without interruption. While in the *ASMR-Break* session, subjects underwent a 4-min ASMR intervention following the initial 15-min PVT, before proceeding to another 15-min PVT. The total duration of PVT was kept the same between two sessions. Electroencephalography (EEG) was recorded throughout each session.

In order to induce the ASMR effect, the audio content was carefully selected, which includes simulated ear-picking sounds (Sakurai et al., 2023), tapping or knocking on wood blocks (Higueras et al., 2023), and whispering by a female voice (Ohta and Inagaki, 2021). These widely-used sounds were chosen based on their effectiveness in eliciting ASMR responses, as documented in prior research (Liu and Zhou, 2019). In the current work, two ASMR audio clips (see Supplementary material for reference), each containing these similar elements, were utilized to ensure consistency and to minimize the impact of any single element on the participants. This approach was aimed at providing a robust ASMR experience while controlling for variability between clips. Both clips were considered effective in triggering ASMR responses in subjects, as validated by previous studies (Liu and Zhou, 2019). After ASMR stimulation, participants were also asked to provide a self-rating of the perceived ASMR intensity. The Short Stress State Questionnaire (SSSQ) was used to assess the mental states before and after each session. The SSSQ consists of 24 items categorized into three subjective experiences: engagement, distress, and worry.

### 2.3 Data acquisition and preprocessing

EEG data were collected using a 64-channel wireless EEG device (Model: NeuSen W64, Neuracle, China) with the placement according to the international 10–20 system. The reference electrodes were positioned between CPZ and CZ, while the sampling rate was set at 1,000 Hz. During data collection, the impedance of each electrode was maintained below 10 kΩ and a 50 Hz notch filter was employed to avoid main interference. During the data collection, participants were instructed to maintain comfortable sitting position to mitigate potential muscular noise in EEG.

Then a previously-validated standard EEG signal preprocessing pipeline was employed (Dimitrakopoulos et al., 2018). First, the raw data were down-sampled to 500 Hz to reduce data volume and computational load. Then, a 1–40 Hz band-pass filter was applied to remove low-frequency drift (e.g., baseline shifts from environmental or physiological factors) and high-frequency noise such as muscle artifacts. Channels with extremely poor signal-to-noise ratio—often due to loose or detached electrodes—were excluded and interpolated using spherical spline interpolation. All signals were then re-referenced to the average of all electrodes to reduce reference bias. Independent component analysis (ICA) was subsequently performed to identify and remove common artifacts, including eye blinks, muscle activity, and cardiac signals. Specifically, ocular artifacts were characterized by large-amplitude components with broad spectral range; muscle artifacts were identified as sharp, high frequency activity (typically > 25 Hz) originating from scalp muscles; and electrocardiographic artifacts were recognized by their stereotyped, high-frequency waveforms resembling ECG morphology.

The EEG data were then segmented into 6-min bins with 50% overlap, leading to each segment containing about 60 trials. Similar to our previous work (Sun et al., 2014), trials with reaction times (RTs) exceeding 500 ms were considered errors, while trials with RTs below 100 ms were considered false alarms. Thus, only trials with reaction times falling within the range of 100–500 ms were included in subsequent analysis. Furthermore, data from a time window of 500 ms following the occurrence of each stimulus were selected for power analysis and brain network construction. Data preprocessing was performed using in-house scripts and the EEGLAB toolbox in Matlab 2021a (MathWorks Inc., USA; Delorme and Makeig, 2004).

In our previous studies (Gao et al., 2020; Sun et al., 2017), we found mixed findings of the recovery effect of in-between task break. Specifically, a mid-task rest break would lead to beneficial effect toward the end of the task (Sun et al., 2017) while transit effect was revealed immediately after a mid-task exercise break (Gao et al., 2020). Therefore, we set out to comprehensively examine the recovery effect of ASMR from both transit and long-term perspectives. Specifically, data from the segments at the beginning of the task (i.e., defined as T1 hereafter) and the end of the task (defined as T4 hereafter) that were corresponding the most vigilant and fatigued state were utilized to investigate the long-term recovery effect of ASMR. Then, the segments immediately before and after the ASMR break were defined as T2 and T3 respectively to reveal the transit recovery effect. In Figure 1, we showed the schematic diagram of the experiment and the corresponding assignment of the EEG electrodes.

### 2.4 Power spectral density

Within each 500 ms epoch, the preprocessed EEG data was segmented using a 50% Hamming window of 250 ms and the Welch method was employed to estimate the power spectral density (PSD) in the conventional canonical frequency bands: delta (1–4 Hz), theta (4–7 Hz), alpha (8–13 Hz), and beta (13–30 Hz). Relative power within each channel was computed as the ratio of the power in a specific band to the total PSD estimated across the range of 1–30 Hz. The averaged relative power for each 6-min segment was obtained from all included epochs within T1, T2, T3, and T4. Previous research had indicated that spectral EEG activity in frontal, central, and posterior cortical regions correlate with mental fatigue (Tran et al., 2020). Therefore, in this study, all recorded electrodes were categorized into these three regions to better investigate the recovery effects of ASMR on mental fatigue. The specific electrode assignment was showed in Figure 1c.

### 2.5 Functional connectivity

In this work, functional connectivity was estimated using the weighted phase lag index (wPLI; Vinck et al., 2011). wPLI is an extension of the PLI (Stam et al., 2007), designed to address certain limitations (i.e., volume conduction and common sources of noise) in capturing true synchronization between brain regions. Heuristically, wPLI calculates the consistency of phase differences between EEG signals recorded from different brain regions and can be estimated as follows:

Let *x*_*j*_(*t*) represents the time series of the *j*^*th*^ channel, then the analytic signal *X*_*j*_(*t*) is computed as:


(1) $$\begin{array}{l} X_{j}\left(t\right)=x_{j}\left(t\right)+i{\bar{x}}_{j}\left(t\right), \end{array}$$

where ${\bar{x}}_{j}\left(t\right)$ denotes the Hilbert transform of *x*_*j*_(*t*) and *i* is the imaginary unit. Let *X*_*k*_(*t*) denotes the analytical signal of the *k*^*th*^ channel, the complex-valued cross-spectrum between two channels can be denoted as:


(2) $$\begin{array}{l} Z\left(t\right)=X_{j}\left(t\right)X_{k}{\left(t\right)}^{*}, \end{array}$$

where $X_{k}{\left(t\right)}^{*}$ denotes the complex conjugate of *X*_*k*_(*t*). Then wPLI is calculated as:


(3) $$\begin{array}{l} wPLI=\frac{\left|\langle ℑ\left(z\right)\rangle \right|}{\langle ℑ\left(z\right)\rangle }=\frac{\left|\langle |ℑ\left(z\right)|sign\left(ℑ\left(z\right)\right)\rangle \right|}{\langle \left|ℑ\left(z\right)\right|\rangle }, \end{array}$$

where ℑ(*z*) denotes the imaginary component of Z, |·| and 〈·〉 represent the absolute and mean value operations respectively, and sign is the signum function. wPLI values vary between 0 and 1, i.e., a value of 0 indicates absence of phase synchronization, while a value of 1 denotes complete synchronization between two channels.

To obtain frequency-specific brain network characteristics, we band-pass filtered EEG data into four canonical frequency bands. Subsequently, for each frequency band, we computed the wPLI for all electrode pairs in each epoch ([0, 500] ms). This approach allowed us to derive an adjacency matrix of size 59 × 59 for each epoch. These adjacency matrices were then averaged across epoch for each segment (i.e., T1, T2, T3, and T4). Therefore, for each participant, there were four 59 × 59 adjacency matrices per segment.

### 2.6 Graph theoretical analysis

To quantitatively assess the changes induced by ASMR on the brain network reorganization, we employed graph theoretical analysis to compare the brain network properties at both global and nodal levels. Specifically, characteristic path length (*L*) and global efficiency (*E*_*glob*_) were utilized to quantify network integration, while clustering coefficient (*C*) and local efficiency (*E*_*loc*_) were employed to measure network segregation. Additionally, the small-worldness index (σ) was used to characterize the balance between network segregation and integration. Of note, σ is the ratio of the clustering coefficient to the path length after both metrics (*C* and *L*) have been standardized through normalizing their values by those of equivalent random networks (Humphries et al., 2006): σ = (*C/C*_*rand*_)*/*(*L/L*_*rand*_). *C*_*rand*_ and *L*_*rand*_ represent the mean clustering coefficient and characteristic path length of 100 matched random networks according to Maslov and Sneppen (2002). At the nodal level, nodal strength (*Str*) was estimated at the weighted network to reveal the nodal characteristics. Here, graph theoretical analysis was performed using the Brain Connectivity Toolbox (Rubinov and Sporns, 2010). For further details pertaining to the formulations and descriptions of these network metrics could be found in our previous work (Sun et al., 2017) or review in the field (Rubinov and Sporns, 2010).

It is noteworthy mentioning that a widely-used sparsity (i.e., the ratio between the number of existing edges to the number of maximum possible edges) approach was initially applied to remove the spurious connections. In this work, a wide sparsity range of [0.1, 0.4] with a step of 0.01 was adopted. The abovementioned global and nodal network metrics were estimated at each sparsity value. To avoid multiple comparisons at the individual sparsity threshold value and to reduce the dependency of statistical results on the arbitrary choice of a single threshold, a previously-validated integration metric was further estimated that was equivalent to the area under the curve of the network metrics (Achard and Bullmore, 2007). The integrated values were set as input for the following statistical analysis.

### 2.7 Statistical analysis

Prior to the statistical analysis, normality was assessed using the Shapiro–Wilk test for all variables. When normality was satisfied (*p* > 0.05), parametric paired-sample *t*-tests were conducted. If normality was not satisfied (*p* < 0.05), non-parametric Wilcoxon matched-pairs signed rank tests were used instead. For within-subject 2 × 2 comparisons, two-way repeated-measures ANOVAs were applied. Specifically, subjective feelings as assessed by the SSSQ scores were quantitatively compared using two-way repeated measures ANOVA with factor #1 time (i.e., before and after PVT) and factor #2 session (i.e., *No-break v.s. ASMR-break*). Of note, separate repeated measures ANOVA was performed for three SSSQ categories (i.e., engagement, distress, and worry). Then, power spectral intensity and brain network metrics were analyzed using two-way repeated measures ANOVA with different factors (i.e., factor #1: time, T1 *vs*. T4 in the general effect whereas T2 *vs*. T3 in the immediate effect; factor #2: session, *No-break vs. ASMR-break*). In addition, four paired-samples *t*-tests (No-break T1 vs. No-break T2; No-break T1 vs. ASMR-break T1; ASMR-break T1 vs. ASMR-break T2; No-break T2 vs. ASMR-break T2) were conducted with 95% confidence intervals. To correct for multiple comparisons in the *t*-test results, the false discovery rate was controlled using the Benjamini–Hochberg procedure, with a threshold of *q* = 0.05 applied to the resulting *p*-values. This correction was also applied to all node-level and connectivity-based *p*-values. Analyses focused on within band changes; consequently, graph metrics were not directly compared across different bands. Spearman's rank correlation coefficient was employed to evaluate the relationship between the changes of behavioral reaction times (ΔRTs) and network metrics (Δnetwork metrics). In order to limit the number of relationship assessments, only global network metrics with significant main effect was included for the association analysis. Statistical analysis for this study was conducted using SPSS software version 26 (IBM, New York), with statistical significance set at *p* < 0.05.

## 3 Results

### 3.1 Behavioral performance

**Figure 2:**
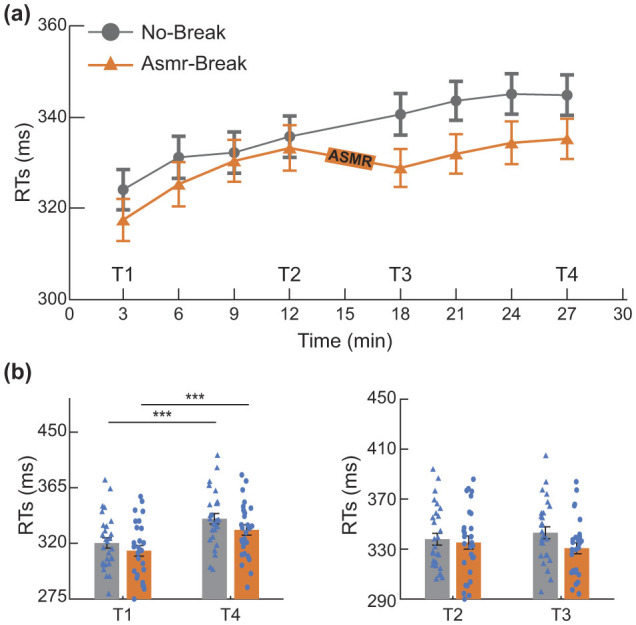
Behavioral performance. **(a)** Reaction times (RTs) during the PVT, binned into sliding 6-min segments (50% overlap) to assess performance fluctuations over time. **(b)** Evaluation of the general and immediate effects of the ASMR intervention. *p < 0.05; ***p < 0.001. Error bars represent the mean ± SEM.

We first examined changes in subjective feelings before and after the PVT. Significant main time effect (*F*_1,27_ = 5.250, *p* = 0.030, η^2^ = 0.163) was revealed in the Engagement. Further *post-hoc* analysis showed the main time effect was attributed to a significant decrease of Engagement in the *No-break* session (*t*_1,27_ = 2.536, *p*_*F*__*DR*_ = 0.030, *Cohen*′*s* d = 0.479), indicating the PVT paradigm led to successful subjective experience of mental fatigue. We then quantitatively compared the behavioral metrics (Figure 2). As for the immediate effect (T2 *vs*. T3), a significant main time-by-session interaction effect was revealed (*F*_1, 27_ = 6.225, *p* = 0.020, η^2^ = 0.187). In terms of the general effect (T1 *vs*. T4), significant main time effect (*F*_1,27_ = 52.047, *p* < 0.001, η^2^ = 0.658) and session effect (*F*_1,27_ = 4.445, *p* = 0.044, η^2^ = 0.141) were revealed. Specifically, in comparison with a monotonically increase pattern of RT in the *No-break* session, the ASMR related recovery revealed at T3 appeared to maintain toward the end of experiment, leading to a marginally significant decrease of RT at T4 (*t*_1,27_ = 2.172, *p*_*FDR*_ = 0.072, *Cohen*′*s* d = 0.410).

To assess whether individual differences in ASMR sensitivity or sex influenced the behavioral outcomes, repeated-measures ANCOVAs were conducted with both self-reported ASMR intensity and sex included as covariates. For the general effect, neither the main effect of ASMR sensitivity (*F*_1,27_ = 0.081, *p* = 0.778, η^2^ = 0.003) nor sex (*F*_1,27_ = 0.107, *p* = 0.747, η^2^ = 0.004) was significant. No significant interactions involving sensitivity or sex were observed (all *p* > 0.1). Similarly, for the immediate effect, the main effects of ASMR sensitivity (*F*_1,27_ = 0.003, *p* = 0.955, η^2^ = 0.000) and sex (*F*_1,27_ = 0.746, *p* = 0.396, η^2^ = 0.028) were both non-significant, and all associated interactions were also non-significant (all *p* > 0.2). These results suggest that neither ASMR sensitivity nor sex had a measurable impact on the primary behavioral findings.

### 3.2 ASMR-related alterations in PSD

**Figure 3:**
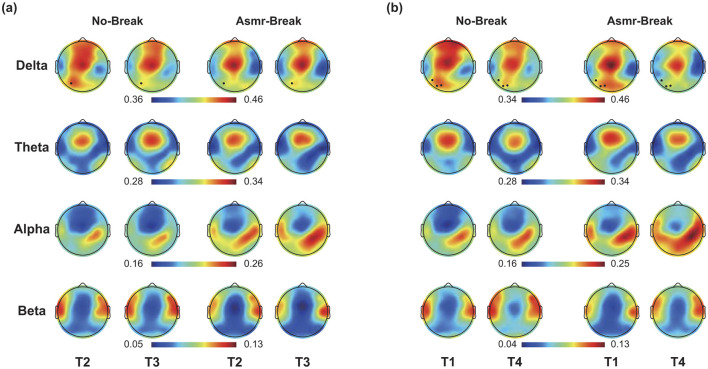
**(a)** Immediate effect (T2 vs. T3) and **(b)** General effect (T1 vs. T4) of ASMR on EEG PSD in four frequency bands. Black dots indicate EEG channel with a significant time-by-session interaction effect.

In Figure 3, we showed the ASMR-related alterations in PSD. Distinct spatio-spectral pattern was revealed for the immediate and general effect. Specifically, we found significant time-by-session interaction in one delta-band EEG channel (Figure 3a) for the immediate effect. As for the general effect, we found the EEG channels with significant interaction was in delta band (*n* = 3) with a left parietal region predilection (Figure 3b). We then interrogated the ASMR-related alterations in different brain regions (Table 1). In terms of the immediate effect, we found significant interaction effect in the parietal region of theta band (*F*_1,27_ = 7.454, *p* = 0.011, η^2^ = 0.216), central region of alpha band (*F*_1,27_ = 6.298, *p* = 0.018, η^2^ = 0.189), and frontal region of beta band (*F*_1,27_ = 8.501, *p* = 0.007, η^2^ = 0.239); whereas significant main session effect was only revealed in the central region of alpha band (*F*_1,27_ = 4.674, *p* = 0.040, η^2^ = 0.148). Main time effect failed to pass the significance threshold. As for the general effect, significant time effect (*p* < 0.05) was revealed in most of the regions across four frequency bands except the parietal regions in theta band (*F*_1,27_ = 1.851, *p* = 0.185, η^2^ = 0.064). Specifically, with the time-on-task increase, a decrease in delta and theta with an increase in alpha and beta power was observed. Comprehensive details of the *post-hoc* statistical analyses are provided in the Supplementary material.

**Table 1:** Statistical results of PSD at different regions.

**Band**	**Region**	**Immediate effect [F(** ***p*** **-value)]**			**General effect [F(** ***p*** **-value)]**		
		**Time**	**Session**	**Interaction**	**Time**	**Session**	**Interaction**
Delta	Frontal	0.157 (0.695)	1.123 (0.299)	1.862 (0.184)	**6.561 (0.016)** ↑	2.683 (0.113)	0.311 (0.581)
	Central	0.329 (0.571)	1.732 (0.199)	0.083 (0.776)	**4.828 (0.037)** ↑	1.235 (0.276)	1.118 (0.300)
	Parietal	2.860 (0.102)	0.300 (0.589)	2.867 (0.102)	**8.853 (0.006)** ↑	0.069 (0.795)	1.353 (0.255)
Theta	Frontal	0.157 (0.695)	0.061 (0.807)	1.663 (0.208)	**5.206 (0.031)** ↑	0.691 (0.413)	0.001 (0.977)
	Central	0.052 (0.822)	0.111 (0.741)	3.686 (0.065)	**8.991 (0.006)** ↑	0.011 (0.919)	0.502 (0.485)
	Parietal	0.014 (0.908)	0.413 (0.708)	**7.454 (0.011)**	1.851 (0.185)	0.012 (0.915)	0.462 (0.503)
Alpha	Frontal	1.729 (0.200)	3.015 (0.094)	0.804 (0.378)	**7.145 (0.013)** ↓	3.314 (0.080)	0.058 (0.811)
	Central	0.282 (0.599)	**4.674 (0.040)** ∇	**6.298 (0.018)**	**5.042 (0.033)** ↓	4.139 (0.052)	0.775 (0.387)
	Parietal	4.188 (0.051)	2.521 (0.124)	0.130 (0.721)	**4.913 (0.035)** ↓	1.458 (0.238)	2.943 (0.098)
Beta	Frontal	2.174 (0.152)	0.193 (0.664)	**8.501 (0.007)**	**12.739 (0.001)** ↓	0.074 (0.788)	1.825 (0.188)
	Central	0.008 (0.930)	0.612 (0.441)	0.342 (0.564)	**9.832 (0.004)** ↓	0.683 (0.416)	0.005 (0.944)
	Parietal	0.024 (0.879)	0.468 (0.389)	1.436 (0.241)	**10.493 (0.003)** ↓	1.040 (0.317)	0.061 (0.806)

Separate two-way repeated-mean ANOVA was performed for immediate effect (T2 vs. T3) and general effect (T1 vs. T4). Significant effects were shown in bold. ∇, No-Break < ASMR-Break; ↑, T1 > T4; ↓, T1 < T4.

### 3.3 ASMR effect on network reorganization

**Figure 4:**
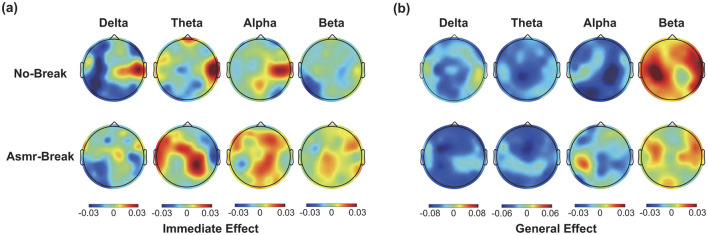
The spatial distribution of **Δ*Str*** (absolute differences) across four frequency bands in this figure **(a)** immediate effect (Δ*Str* = *Str*_T3_ –* Str*_T2_) and **(b)** general effect Δ*Str* = *Str*_T4_ –* Str*_T1_. Black dots indicate nodes with significant time-by-session interaction effect.

We then looked into the ASMR effect on brain network reorganization. In Table 2, we showed the statistical results of network metrics. In terms of immediate effect, only σ in theta band exhibited a significant time-by-session interaction effect (*F*_1,27_ = 7.189, *p* = 0.012, η^2^ = 0.210). Following *post-hoc* analysis suggested that the significant interaction effect was attributed to a significant increase of σ from T2 to T3 in the *No-break* session compared to a non-significant decrease in the *ASMR-break* session (*t*_1,27_ = 2.473, *p*_*FDR*_ = 0.040, *Cohen*′*s* d = 0.467). Main time and session effect failed to pass the significance threshold. As for the general effect, significant time effect was observed in network metrics in delta, theta and alpha band (Table 2). Further inspection of the main time effect, we found a less optimal network topology as shown in reduced local segregation as well as global integration toward the end of task. Detailed *post-hoc* analyses of the statistical results could be found in Supplementary material. In Figure 4, we showed the nodal characteristics. However, no EEG channel exhibited a significant time-by-session interaction for either the immediate or general effect.

**Table 2:** Statistical results of network metrics.

**Band**	**Parameter**	**Immediate effect [F(** ***p*** **-value)]**			**General effect [F(** ***p*** **-value)]**		
		**Time**	**Session**	**Interaction**	**Time**	**Session**	**Interaction**
Delta	*C*	0.026 (0.762)	0.018 (0.895)	0.094 (0.762)	3.147 (0.087)	0.049 (0.827)	1.504 (0.231)
	*L*	3.043 (0.092)	0.089 (0.767)	0.701 (0.410)	**10.409 (0.003)** ↓	0.711 (0.407)	1.704 (0.203)
	σ	1.615 (0.215)	0.484 (0.492)	0.726 (0.402)	**6.238 (0.019)** ↓	0.275 (0.604)	1.326 (0.260)
	*Eglob*	1.186 (0.286)	0.166 (0.687)	0.361 (0.553)	**8.363 (0.007)**↑	0.211 (0.650)	1.817 (0.189)
	*Eloc*	0.358 (0.555)	0.001 (0.992)	0.069 (0.794)	**5.928 (0.022)**↑	0.345 (0.562)	1.546 (0.224)
Theta	*C*	0.023 (0.879)	0.227 (0.637)	0.508 (0.482)	**16.044 (**<**0.001)**↑	0.350 (0.559)	0.214 (0.648)
	*L*	0.228 (0.637)	1.040 (0.317)	0.056 (0.815)	**14.738 (0.001)**↓	0.972 (0.333)	0.236 (0.631)
	σ	0.301 (0.587)	2.479 (0.127)	**7.198 (0.012)**	3.166 (0.086)	1.824 (0.188)	0.392 (0.537)
	*Eglob*	0.022 (0.883)	0.238 (0.630)	0.001 (0.971)	**14.821 (0.001)**↑	1.640 (0.211)	0.046 (0.831)
	*Eloc*	0.002 (0.962)	0.093 (0.763)	0.597 (0.447)	**15.947 (**<**0.001)**↑	0.885 (0.355)	0.031 (0.861)
Alpha	*C*	0.003 (0.959)	0.458 (0.504)	0.380 (0.543)	1.361 (0.254)	0.054 (0.819)	3.121 (0.089)
	*L*	1.280 (0.268)	0.653 (0.426)	0.250 (0.621)	2.144 (0.155)	0.000 (0.985)	1.499 (0.231)
	σ	0.779 (0.385)	0.035 (0.853)	0.025 (0.875)	**4.285 (0.048)**↓	0.062 (0.805)	0.779 (0.385)
	*Eglob*	0.675 (0.419)	0.234 (0.633)	0.012 (0.915)	2.490 (0.126)	0.001 (0.974)	1.787 (0.193)
	*Eloc*	0.104 (0.749)	0.127 (0.725)	0.196 (0.662)	1.704 (0.203)	0.028 (0.868)	2.276 (0.143)
Beta	*C*	0.012 (0.912)	0.114 (0.738)	0.464 (0.501)	3.450 (0.074)	0.005 (0.944)	0.899 (0.352)
	*L*	0.130 (0.721)	0.105 (0.749)	0.662 (0.423)	3.378 (0.077)	0.003 (0.959)	2.125 (0.156)
	σ	0.852 (0.364)	0.044 (0.835)	0.018 (0.895)	1.698 (0.204)	0.012 (0.915)	0.089 (0.767)
	*Eglob*	0.006 (0.941)	0.001 (0.978)	1.562 (0.222)	3.827 (0.061)	0.000 (0.985)	0.780 (0.385)
	*Eloc*	0.001 (0.977)	0.054 (0.818)	1.474 (0.235)	3.998 (0.056)	0.001 (0.971)	0.744 (0.396)

Separate two-way repeated-mean ANOVA was performed for immediate effect (T2 vs. T3) and general effect (T1 vs. T4). Significant effects were shown in bold; ↑, T1 > T4; ↓, T1 < T4.

### 3.4 Correlation

**Figure 5:**
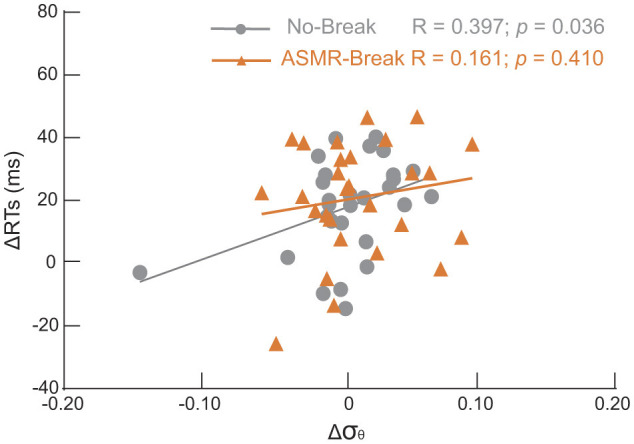
Scatter plots of changes of small-worldness (Δσ) in theta band between T4 and T1 and the corresponding changes of reaction time (Δ*RT*). Significantly positive correlation was revealed only in the *No-break* session (gray circles); that is the larger RT increment the higher Δσ_θ_. Such significant association was absent in the *ASMR-rest* session.

For those global network metrics that exhibited significant main effect, only small-worldness in theta band of No–break session was significantly correlated with behavioral performance for the general effect (Figure 5). That is, Δσ_θ_ was significantly correlated with ΔRT in the No–break session (R = 0.397, *p* = 0.036), indicating brain network tends to become more small-world toward the end of task to compensate the behavioral deterioration. The significant correlation was absent in the ASMR – break session (*R* = 0.161, *p* = 0.410).

## 4 Discussion

In this study, we examined the restorative effects of ASMR on mental fatigue and delved into the underlying neural mechanisms facilitating this recovery. Our key findings are as follows: first, ASMR demonstrated an immediate restorative effect on behavioral performance, though general beneficial effects were not observed. Second, significant EEG power alterations were noted following ASMR exposure, notably on P_θ___parietal_, P_α___central_, and P_β___frontal_. Particularly, a significant decrease in P_β___frontal_ was observed during the *ASMR-Break* session. Third, a significant interaction effect was observed in the small-worldness within theta band, suggesting an immediate restorative effect of ASMR. Fourth, during the *No-Break* session, changes of RT were significantly positively correlated with the σ in the theta band, but this correlation was absent in the *ASMR-Break* session. These findings are discussed in greater detail below.

### 4.1 Behavioral effect of ASMR

According to the resource model theory (Boksem and Tops, 2008), prolonged tasks deplete cognitive resources, leading to the time-on-task effect, which manifests as a performance decline (Liu and Zhang, 2023). Consistent with previous fatigue studies, behavioral performance significantly deteriorated after performing continuous PVT (Dai et al., 2022). Introducing ASMR during tasks revealed significant interactions in the immediate effect analysis. As noted by Barratt and Davis (2015), ASMR induces a distinct, flow-like mental state that enhances relaxation and enriches the subjective feeling of pleasure and calmness (Inagaki and Ohta, 2022). This mental state may help reduce mental workloads. Moreover, ASMR has been reported to be associated with reduced heart rate, which is indicative of relaxation (Poerio et al., 2018). This effect is supported by other research (Lohaus et al., 2023) showing that ASMR induces a higher state of relaxation in comparison with pure rest. Consistently, we observed that the significant decrease in engagement and increase in distress typically caused by continuous PVT were mitigated following the ASMR intervention. However, we did not observe a significant interaction effect concerning the general effect. At the first blush, this may seem counterintuitive especially when significant beneficial effect was observed for the immediate effect. Of note, there have been several instances where the recuperative effect of a mid-task break on task performance has not been observed (Lim and Kwok, 2016; Sun et al., 2017). In fact, in our previous work where a 5-min mid-task rest was administered to provide recovery effect of mental fatigue (Sun et al., 2017). Similar to the current work, we failed to reveal the recuperative effect on behavioral performance. In line with these findings, Lim and colleagues showed that taking a longer break leads to higher immediate rebound in performance yet greater subsequent TOT decline, pointing an important moderator of break duration on the fatigue recovery (Lim and Kwok, 2016). The duration–effect principle also applies to ASMR: Most ASMR sensitive individuals report tingles and relaxation within the first 2–4 min of a video (Valtakari et al., 2019). Another study showed that around 3 min of ASMR viewing led to decreases in heart rate and increases in skin conductance, indicating an ASMR state. Tingle intensity and subjective relaxation ratings increased with 5–10 min of exposure compared to shorter viewing times (Engelbregt et al., 2022; Terashima et al., 2024). Additionally, longer ASMR durations significantly shorten sleep latency (Wu et al., 2024). Another key moderator is the type of break. We previously reported that a mid-task 15-min cycling exercise would lead to both immediate and general recuperative effect (Gao et al., 2020). In sum, recovery of cognitive performance following a mid-task break is not a given and is heavily relied on the characteristics of the break (including duration, types, and administration time), highlighting the need for caution in interpreting these results and more importantly in practical applications for fatigue recovery.

### 4.2 EEG power changes induced by ASMR

In terms of the underlying neural mechanisms, we first interrogated the spatio-spectral alterations. Immediately following the ASMR intervention, there was a clear modulation in the power spectral densities across several frequency bands. Specifically, notable EEG changes immediately following ASMR exposure included a decrease in beta power in frontal regions, along with alterations in theta and alpha bands across various cortical areas. These alterations suggest a complex interplay between ASMR stimuli and brain electrophysiological responses, impacting cognitive states and fatigue levels. Previous studies have indicated that ASMR may reduce beta activity (Inagaki and Ohta, 2022). Increased beta activity is often reported in state of mental fatigue (Craig et al., 2012), which is associated with heightened alertness (Kamiński et al., 2012) and focused attention (Palacios-García et al., 2021). And beta-gamma coupling in the frontal was observed during driving fatigue (Liu et al., 2023). Therefore, ASMR stimuli may promote a shift from states of heightened alertness to a more balanced neural oscillatory profile, thereby supporting recovery of cognitive resources. Alpha waves, typically associated with wakeful relaxation, may reflect a calm but alert state (Lombardi et al., 2023). Previous research indicates that alpha power increases in states of mental fatigue (Magnuson et al., 2021), aiding in attention maintenance by suppressing distracting inputs (Zhao et al., 2023). Recent EEG-based neurometrics have demonstrated that the alpha-based Mental Drowsiness index can reliably detect fatigue during driving in real time (Ronca et al., 2022; Giorgi et al., 2023). ASMR has been shown to trigger increased alpha wave activity (Fredborg et al., 2021), which is linked to the relaxation. Therefore, the enhancement of alpha activity due to ASMR in our study is more likely indicative of relaxation, rather than a direct response to mental fatigue or functional inhibition (Fredborg et al., 2021). Theta activity has consistently been reported as a reliable indicator of mental fatigue (Cao et al., 2014; Wascher et al., 2014; Brietzke et al., 2021), with its enhancement associated with poorer performance in tasks requiring sustained attention (Kao et al., 2013). Contrary to the traditional view that links theta activity to increased task engagement (Onton et al., 2005; Smit et al., 2005). The enhancement of theta activity contradicts the core idea that it is associated with an increasing reluctance to further engage in tasks during the process of mental fatigue (Grandjean, 1979; Tops and Boksem, 2010). Moreover, studies utilizing event-related potential (ERP) analysis methods consistently show that as task duration increases, the amplitude of theta-related ERPs declines (Guo et al., 2016; Hopstaken et al., 2015; Möckel et al., 2015). Our findings also show a significant reduction in theta as mental fatigue progresses, possibly because the time window we captured is the stimulus window. Evidence suggests that while theta activity may increase during inter-trial intervals in states of fatigue, task-related theta activity diminishes (Arnau et al., 2021). Another study indicated that theta activity in the parietal region was linked to attention (Yang et al., 2017). Similar to the alpha band, the observed reduction in theta due to ASMR may stem from its relaxing effects. As for the general effect, a systematic change is evident, typically characterized by a decrease in delta and theta power alongside an increase in alpha and beta power. These trends are consistent with prior observations that mental fatigue is accompanied by shifts in spectral power (Craig et al., 2012; Magnuson et al., 2021; Möckel et al., 2015; Yirikogullari et al., 2025). Although the behavioral analysis indicates that ASMR did not completely eliminate the accumulation of fatigue, the fatigue-related spectral changes were partially mitigated in the ASMR session. For example, the significant increase in P_β__frontal_ from T1 to T4 was observed only in the No-Break session, suggesting that ASMR intervention may help curb the up-regulation of beta activity that is typically associated with heightened alertness or compensatory efforts under fatigue. Overall, the EEG power dynamics alterations observed in response to ASMR interventions underscore its potential role in modulating brain function to enhance relaxation and cognitive efficiency. By increasing alpha power, ASMR seems to promote a relaxed state that aids in maintaining focus amidst mental fatigue, while decreases in beta and theta power might lessen the overall cognitive load and reduce active engagement in demanding tasks. These insights expand our understanding of ASMR's benefits beyond mere relaxation, suggesting its utility in fostering cognitive and psychological resilience in high-demand scenarios.

### 4.3 Network alterations induced by ASMR

In comparison with the widely-investigated spectrum alterations of mental fatigue and fatigue recovery, effects of fatigue recovery on brain network reorganization are less understood (Qi et al., 2019). Here, we reported the topological reorganizations of brain network due to ASMR modulation. In terms of immediate effects, our results indicate that ASMR significantly counteracts the increase in σ within the theta band. This immediate modulation appears to improve cognitive resilience and improve performance in tasks that require sustained attention. By stabilizing small-worldness, ASMR may help preserve the balance between local specialization and global integration within brain networks, which is essential for optimal cognitive functioning (Pisarchik et al., 2023). This finding suggests that ASMR could play a crucial role in maintaining efficient brain network dynamics under fatigue-inducing conditions. When considering the general effect, mental fatigue is marked by the significant time effect, that is, network reorganization within the delta and theta band. This reorganization is characterized by notable decreases in both global and local efficiency, alongside a significant increase in characteristic path length, together suggesting a comprehensive decline in network connectivity efficiency. Previous research has reported similar increase trend in the characteristic path length (Peng et al., 2022) as well as reduced global efficiency and increased local clustering (Giannakopoulou et al., 2023) under fatigue. Notably, although ASMR did not completely prevent these general network changes, it did disrupt the correlation between the σ and RTs. The disruption of the σ-RTs correlation further supports the potential of ASMR to mitigate the effects of fatigue, enabling individuals to sustain attention and performance levels over extended periods. These insights provide a promising approach for enhancing cognitive resilience and performance in high-demand environments.

### 4.4 Methodological considerations

Several issues need to be acknowledged when interpreting our findings. First, a within-subject design was employed in this work to explore the effect of ASMR for mental fatigue. Although this could be an advantage to account for the well-known inter-individual differences (Sun et al., 2014), the sample size was relatively small and primarily composed of healthy college students, which limits the generalizability of our conclusions. As our work is one of the first exploratory studies to investigate the restorative effect of ASMR on fatigue recovery, future research with larger, more diverse cohorts—including a broader age range—is needed to validate and extend our findings. Second, the regulation of ASMR exhibits significant individual differences. Although we carefully screened and selected the audio stimuli based on preliminary evaluations (Sakurai et al., 2023), individual variability in sensitivity and response to ASMR may still influence the outcomes (Poerio et al., 2022) and complicated the interpretation of our results. Investigating how neuro-diversity or other psychological factors modulate ASMR responsiveness will be a crucial step in understanding its full potential. Finally, the ASMR intervention in this study was set at a duration of 4 min, while previous research suggests that the duration of an intervention can significantly affect fatigue recovery. However, Lim and colleagues found that taking a longer break leads to greater immediate redound in performance yet greater subsequent TOT decline (Lim and Kwok, 2016) that might be attributed to motivation switch. Moreover, we have previously investigated the effect of mid-task break on fatigue recovery with various durations [i.e., 5.4 min rest break (Sun et al., 2017), 15 min exercise break (Gao et al., 2020)]. Divergent findings were revealed, highlighting the equivalent importance of both the content and length of the break on fatigue recovery. Future studies should explore various types and durations of ASMR stimulation to determine the optimal parameters for fatigue alleviation in practical applications.

## 5 Conclusion

In conclusion, this study explored ASMR's neural mechanisms in alleviating mental fatigue through behavioral and electrophysiological assessments. Our findings confirm that ASMR provides immediate relief from mental fatigue by interacting with the brain's networks, not just by countering fatigue directly. This research underscores ASMR's potential as a therapeutic tool in high-demand settings and sets the stage for further exploration of non-pharmacological interventions to enhance mental resilience and performance.

## Data Availability

The original contributions presented in the study are included in the article/Supplementary material, further inquiries can be directed to the corresponding authors.
