# Supplementary material for: Effects of ASMR on mental fatigue recovery revealed by EEG power and brain network analysis
Source: Front Hum Neurosci. 2025 Jul 16;19:1619424. doi: 10.3389/fnhum.2025.1619424 (PMC12307496; doi:10.3389/fnhum.2025.1619424)
Supplement: Supplementary file 1 [file Table_1.docx]

Supplementary Material

# Supplementary Tables

TABLE I．Statistical Results of Immediate Effect on PSD at different region regions

| Band | Region | Immediate Effect (F(p-value)) | | |  | Post-hoc (t (p-value)) | | | |
| --- | --- | --- | --- | --- | --- | --- | --- | --- | --- |
|  |  | Time | Session | Interaction |  | *N_T_* _2_ *vs. A_T_* _2_ | *N_T_* _3_ *vs. A_T_* _3_ | *N_T_* _2_ *vs. N_T_* _3_ | *A_T_* _2_ *vs. A_T_* _3_ |
| Delta | Frontal | 0.157 (0.695) | 1.123 (0.299) | 1.862 (0.184) |  | 1.697 (0.339) | 0.365 (0.718) | 1.412 (0.339) | 0.593 (0.718) |
|  | Central | 0.329 (0.571) | 1.732 (0.199) | 0.083 (0.776) |  | 1.033 (0.654 ) | 1.212 (0.654) | 0.171 (0.866) | 0.525 (0.805) |
|  | Parietal | 2.860 (0.102) | 0.300 (0.589) | 2.867 (0.102) |  | 1.189 (0.490) | 0.173 (0.946) | **2.836 (0.034)** | 0.068 (0.946) |
| Theta | Frontal | 0.157 (0.695) | 0.061 (0.807) | 1.663 (0.208) |  | 0.663 (0.817) | 0.234 (0.817) | 1.203 (0.817) | 0.457 (0.817) |
|  | Central | 0.052 (0.822) | 0.111 (0.741) | 3.686 (0.065) |  | 0.407 (0.687) | 0.863 (0.214) | 1.254 (0.307) | 1.228 (0.307) |
|  | Parietal | 0.014 (0.908) | 0.413 (0.708) | **7.454 (0.011)** |  | 0.768 (0.780) | 1.192 (0.325) | 1.625 (0.231) | 1.949 (0.231) |
| Alpha | Frontal | 1.729 (0.200) | 3.015 (0.094) | 0.804 (0.378) |  | 1.337 (0.385) | 1.877 (0.285) | 0.416 (0.681) | 1.592 (0.584) |
|  | Central | 0.282 (0.599) | **4.674 (0.040)** ▽ | **6.298 (0.018)** |  | 1.391 (0.339) | **2.780 (0.044)** | 1.313 (0.339) | 1.829 (0.339) |
|  | Parietal | 4.188 (0.051) | 2.521 (0.124) | 0.130 (0.721) |  | 1.373 (0.229) | 1.647 (0.229) | 1.230 (0.229) | 1.529 (0.229) |
| Beta | Frontal | 2.174 (0.152) | 0.193 (0.664) | **8.501 (0.007)** |  | 0.094 (0.610) | 0.935 (0.662) | 0.494 (0.662) | **2.982 (0.024)** |
|  | Central | 0.008 (0.930) | 0.612 (0.441) | 0.342 (0.564) |  | 0.667 (0.902) | 0.868 (0.902) | 0.274 (0.902) | 0.423 (0.902) |
|  | Parietal | 0.024 (0.879) | 0.468 (0.389) | 1.436 (0.241) |  | 0.694 (0.779) | 1.026 (0.736) | 0.803 (0.314) | 0.802 (0.314) |

Note: Two-way repeated-mean ANOVA was performed for immediate effect. N represents No-break session and A represents ASMR-break session. Significant effects were shown in **bold**; *↑*, T1 *>* T4; *↓*, T1 *<* T4.

TABLE II．Statistical Results of GENERAL Effect on PSD at different region regions

| Band | Region | General Effect (F(p-value)) | | |  | Post-hoc (t (p-value)) | | | |
| --- | --- | --- | --- | --- | --- | --- | --- | --- | --- |
|  |  | Time | Session | Interaction |  | *N_T_* _2_ *vs. A_T_* _2_ | *N_T_* _3_ *vs. A_T_* _3_ | *N_T_* _2_ *vs. N_T_* _3_ | *A_T_* _2_ *vs. A_T_* _3_ |
| Delta | Frontal | **6.561 (0.016)** *↑* | 2.683 (0.113) | 0.311 (0.581) |  | 1.532 (0.183) | 1.342 (0.191) | 2.168 (0.157) | 1.596 (0.183) |
|  | Central | **4.828 (0.037)** *↑* | 1.235 (0.276) | 1.118 (0.300) |  | 0.504 (0.619) | 1.413 (0.338) | 0.961 (0.450) | 2.481 (0.079) |
|  | Parietal | **8.853 (0.006)** *↑* | 0.069 (0.795) | 1.353 (0.255) |  | 0.340 (0.737) | 0.798 (0.576) | 1.459 (0.312) | **2.935 (0.027)** |
| Theta | Frontal | **5.206 (0.031)** *↑* | 0.691 (0.413) | 0.001 (0.977) |  | 0.712 (0.483) | 0.779 (0.483) | 1.672 (0.212) | 2.027 (0.211) |
|  | Central | **8.991 (0.006)** *↑* | 0.011 (0.919) | 0.502 (0.485) |  | 0.346 (0.183) | 0.192 (0.849) | 2.536 (0.069) | 2.378 (0.076) |
|  | Parietal | 1.851 (0.185) | 0.012 (0.915) | 0.462 (0.503) |  | 0.302 (0.902) | 0.125 (0.902) | 0.859 (0.796) | 1.562 (0.520) |
| Alpha | Frontal | **7.145 (0.013)** *↓* | 3.314 (0.080) | 0.058 (0.811) |  | 1.731 (0.127) | 1.617 (0.316) | 1.885 (0.127) | 2.205 (0.127) |
|  | Central | **5.042 (0.033)** *↓* | 4.139 (0.052) | 0.775 (0.387) |  | 1.907 (0.134) | 1.934 (0.146) | 1.134 (0.267) | 2.286 (0.121) |
|  | Parietal | **4.913 (0.035)** *↓* | 1.458 (0.238) | 2.943 (0.098) |  | 0.593 (0.558) | 1.516 (0.407) | 0.868 (0.403) | 2.619 (0.057) |
| Beta | Frontal | **12.739 (0.001)** *↓* | 0.074 (0.788) | 1.825 (0.188) |  | 0.109 (0.421) | 0.720 (0.745) | **3.612 (0.004)** | 1.718 (0.195) |
|  | Central | **9.832 (0.004)** *↓* | 0.683 (0.416) | 0.005 (0.944) |  | 0.791 (0.902) | 0.822 (0.902) | **2.381 (0.049)** | **2.931 (0.027)** |
|  | Parietal | **10.493 (0.003)** *↓* | 1.040 (0.317) | 0.061 (0.806) |  | 0.961 (0.695) | 1.064 (0.499) | **2.207 (0.016)** | **3.775 (***<* **0.001)** |

Note: Two-way repeated-mean ANOVA was performed for general effect. N represents No-break session and A represents ASMR-break session. Significant effects were shown in **bold**; *↑*, T1 *>* T4; *↓*, T1 *<* T4.

TABLE III．Statistical Results of immediate Effect on Network metrics

| Band | Region | Immediate Effect (F(p-value)) | | |  | Post-hoc (t (p-value)) | | | |
| --- | --- | --- | --- | --- | --- | --- | --- | --- | --- |
|  |  | Time | Session | Interaction |  | *N_T_* _2_ *vs. A_T_* _2_ | *N_T_* _3_ *vs. A_T_* _3_ | *N_T_* _2_ *vs. N_T_* _3_ | *A_T_* _2_ *vs. A_T_* _3_ |
| Delta | *C* | 0.026 (0.762) | 0.018 (0.895) | 0.094 (0.762) |  | 0.342 (0.974) | 0.032 (0.974) | 0.046 (0.974) | 0.402 (0.974) |
|  | *L* | 3.043 (0.092) | 0.089 (0.767) | 0.701 (0.410) |  | 0.862 (0.736) | 0.198 (0.845) | 1.776 (0.348) | 0.770 (0.736) |
|  | *σ* | 1.615 (0.215) | 0.484 (0.492) | 0.726 (0.402) |  | 0.946 (0.706) | 0.210 (0.835) | 1.591 (0.493) | 0.213 (0.835) |
|  | *Eglob* | 1.186 (0.286) | 0.166 (0.687) | 0.361 (0.553) |  | 0.746 (0.818) | 0.057 (0.955) | 1.098 (0.818) | 0.511 (0.818) |
|  | *Eloc* | 0.358 (0.555) | 0.001 (0.992) | 0.069 (0.794) |  | 0.160 (0.919) | 0.102 (0.919) | 0.587 (0.919) | 0.376 (0.919) |
| Theta | *C* | 0.023 (0.879) | 0.227 (0.637) | 0.508 (0.482) |  | 0.018 (0.986) | 0.703 (0.986) | 0.552 (0.986) | 0.614 (0.986) |
|  | *L* | 0.228 (0.637) | 1.040 (0.317) | 0.056 (0.815) |  | 0.677 (0.938) | 0.959 (0.938) | 0.098 (0.938) | 0.510 (0.938) |
|  | *σ* | 0.301 (0.587) | 2.479 (0.127) | **7.198 (0.012)** |  | **2.889 (0.030)** | 0.530 (0.600) | **2.474 (0.040)** | 1.766 (0.118) |
|  | *Eglob* | 0.022 (0.883) | 0.238 (0.630) | 0.001 (0.971) |  | 0.450 (0.951) | 0.365 (0.951) | 0.062 (0.951) | 0.138 (0.951) |
|  | *Eloc* | 0.002 (0.962) | 0.093 (0.763) | 0.597 (0.447) |  | 0.192 (0.849) | 0.619 (0.775) | 0.558 (0.775) | 0.693 (0.775) |
| Alpha | *C* | 0.003 (0.959) | 0.458 (0.504) | 0.380 (0.543) |  | 0.908 (0.844) | 0.199 (0.844) | 0.357 (0.844) | 0.512 (0.844) |
|  | *L* | 1.280 (0.268) | 0.653 (0.426) | 0.250 (0.621) |  | 0.463 (0.647) | 1.015 (0.647) | 0.996 (0.647) | 0.547 (0.647) |
|  | *σ* | 0.779 (0.385) | 0.035 (0.853) | 0.025 (0.875) |  | 0.046 (0.964) | 0.272 (0.964) | 0.522 (0.964) | 0.802 (0.743) |
|  | *Eglob* | 0.675 (0.419) | 0.234 (0.633) | 0.012 (0.915) |  | 0.426 (0.683) | 0.412 (0.683) | 0.460 (0.683) | 0.701 (0.683) |
|  | *Eloc* | 0.104 (0.749) | 0.127 (0.725) | 0.196 (0.662) |  | 0.507 (0.961) | 0.050 (0.961) | 0.082 (0.961) | 0.603 (0.961) |
| Beta | *C* | 0.012 (0.912) | 0.114 (0.738) | 0.464 (0.501) |  | 0.030 (0.999) | 0.641 (0.840) | 0.238 (0.999) | 0.419 (0.999) |
|  | *L* | 0.130 (0.721) | 0.105 (0.749) | 0.662 (0.423) |  | 0.189 (0.852) | 0.945 (0.852) | 0.771 (0.852) | 0.231 (0.852) |
|  | *σ* | 0.852 (0.364) | 0.044 (0.835) | 0.018 (0.895) |  | 0.059 (0.953) | 0.278 (0.953) | 0.803 (0.953) | 0.564 (0.953) |
|  | *Eglob* | 0.006 (0.941) | 0.001 (0.978) | 1.562 (0.222) |  | 0.558 (0.920) | 0.785 (0.765) | 0.649 (0.765) | 0.570 (0.765) |
|  | *Eloc* | 0.001 (0.977) | 0.054 (0.818) | 1.474 (0.235) |  | 0.339 (0.867) | 0.973 (0.480) | 0.598 (0.867) | 0.552 (0.867) |

Note: Two-way repeated-mean ANOVA was performed for immediate effect. N represents No-break session and A represents ASMR-break session. Significant effects were shown in **bold**.

TABLE IV．Statistical Results of GENERAL Effect on Network metrics

| Band | Region | General Effect Effect (F(p-value)) | | |  | Post-hoc (t (p-value)) | | | |
| --- | --- | --- | --- | --- | --- | --- | --- | --- | --- |
|  |  | Time | Session | Interaction |  | *N_T_* _2_ *vs. A_T_* _2_ | *N_T_* _3_ *vs. A_T_* _3_ | *N_T_* _2_ *vs. N_T_* _3_ | *A_T_* _2_ *vs. A_T_* _3_ |
| Delta | *C* | 3.147 (0.087) | 0.049 (0.827) | 1.504 (0.231) |  | 0.761 (0.604) | 0.819 (0.604) | 0.480 (0.635) | **3.257 (0.012)** |
|  | *L* | **10.409 (0.003)** *↓* | 0.711 (0.407) | 1.704 (0.203) |  | 0.178 (0.860) | 1.189 (0.326) | 1.443 (0.321) | **3.953 (0.002)** |
|  | *σ* | **6.238 (0.019)** *↓* | 0.275 (0.604) | 1.326 (0.260) |  | 1.337 (0.284) | 0.272 (0.788) | 1.276 (0.284) | **2.800 (0.037)** |
|  | *Eglob* | **8.363 (0.007)** *↑* | 0.211 (0.650) | 1.817 (0.189) |  | 0.407 (0.687) | 1.040 (0.410) | 1.449 (0.318) | **4.073 (0.001)** |
|  | *Eloc* | **5.928 (0.022)** *↑* | 0.345 (0.562) | 1.546 (0.224) |  | 0.421 (0.677) | 1.058 (0.464) | 0.955 (0.464) | **4.122 (0.001)** |
| Theta | *C* | **16.044 (***<***0.001)** *↑* | 0.350 (0.559) | 0.214 (0.648) |  | 0.248 (0.814) | 0.649 (0.814) | **3.143 (0.024)** | **2.904 (0.024)** |
|  | *L* | **14.738 (0.001)** *↓* | 0.972 (0.333) | 0.236 (0.631) |  | 0.575 (0.630) | 0.899 (0.630) | **3.058 (0.015)** | **3.283 (0.011)** |
|  | *σ* | 3.166 (0.086) | 1.824 (0.188) | 0.392 (0.537) |  | **1.969 (0.033)** | 0.508 (0.615) | 0.898 (0.139) | 1.712 (0.139) |
|  | *Eglob* | **14.821 (0.001)** *↑* | 1.640 (0.211) | 0.046 (0.831) |  | 1.461 (0.207) | 0.831 (0.711) | **2.789 (0.044)** | **3.471 (0.007)** |
|  | *Eloc* | **15.947 (***<***0.001)** *↑* | 0.885 (0.355) | 0.031 (0.861) |  | 0.835 (0.548) | 0.749 (0.902) | 2.938 (0.067) | **3.331 (0.010)** |
| Alpha | *C* | 1.361 (0.254) | 0.054 (0.819) | 3.121 (0.089) |  | 1.230 (0.458) | 0.786 (0.585) | 1.881 (0.282) | 0.202 (0.842) |
|  | *L* | 2.144 (0.155) | 0.000 (0.985) | 1.499 (0.231) |  | 0.659 (0.739) | 0.598 (0.739) | 1.988 (0.228) | 0.251 (0.804) |
|  | *σ* | **4.285 (0.048)** *↓* | 0.062 (0.805) | 0.779 (0.385) |  | 1.094 (0.567) | 0.393 (0.697) | 1.976 (0.234) | 0.770 (0.597) |
|  | *Eglob* | 2.490 (0.126) | 0.001 (0.974) | 1.787 (0.193) |  | 0.767 (0.450) | 0.781 (0.450) | 1.999 (0.223) | 0.457 (0.450) |
|  | *Eloc* | 1.704 (0.203) | 0.028 (0.868) | 2.276 (0.143) |  | 1.079 (0.500) | 0.683 (0.500) | 1.964 (0.240) | 0.090 (0.500) |
| Beta | *C* | 3.450 (0.074) | 0.005 (0.944) | 0.899 (0.352) |  | 0.922 (0.486) | 0.483 (0.420) | 2.491 (0.077) | 0.684 (0.884) |
|  | *L* | 3.378 (0.077) | 0.003 (0.959) | 2.125 (0.156) |  | 0.912 (0.493) | 0.949 (0.275) | 2.333 (0.110) | 0.445 (0.762) |
|  | *σ* | 1.698 (0.204) | 0.012 (0.915) | 0.089 (0.767) |  | 0.068 (0.946) | 0.305 (0.946) | 0.700 (0.946) | 1.233 (0.946) |
|  | *Eglob* | 3.827 (0.061) | 0.000 (0.985) | 0.780 (0.385) |  | 0.768 (0.599) | 0.498 (0.528) | 2.347 (0.106) | 0.800 (0.728) |
|  | *Eloc* | 3.998 (0.056) | 0.001 (0.971) | 0.744 (0.396) |  | 0.786 (0.585) | 0.508 (0.387) | 2.467 (0.081) | 0.810 (0.831) |

Note: Two-way repeated-mean ANOVA was performed for general effect. N represents No-break session and A represents ASMR-break session. Significant effects were shown in **bold**.
